# *Fusobacterium nucleatum* in Colorectal Cancer: Ally Mechanism and Targeted Therapy Strategies

**DOI:** 10.34133/research.0640

**Published:** 2025-04-09

**Authors:** Junna Lu, Wei Wei, Diwei Zheng

**Affiliations:** ^1^ State Key Laboratory of Biopharmaceutical Preparation and Delivery, Institute of Process Engineering, Chinese Academy of Sciences, Beijing 100190, China.; ^2^ School of Chemical Engineering, University of Chinese Academy of Sciences, Beijing 100049, China.

## Abstract

*Fusobacterium nucleatum* (*Fn*), an oral anaerobic commensal, has recently been identified as a crucial oncogenic contributor to colorectal cancer pathogenesis through its ectopic colonization in the gastrointestinal tract. Accumulating evidence reveals its multifaceted involvement in colorectal cancer initiation, progression, metastasis, and therapeutic resistance to conventional treatments, including chemotherapy, radiotherapy, and immunotherapy. This perspective highlights recent advances in anti-*Fn* strategies, including small-molecule inhibitors, nanomedicines, and biopharmaceuticals, while critically analyzing the translational barriers in developing targeted antimicrobial interventions. We further propose potential strategies to overcome current challenges in *Fn* modulation, aiming to pave the way for more effective therapeutic interventions and better clinical outcomes.

## *Fusobacterium nucleatum* as an “Ally” in Colorectal Cancer

### Pathological properties of colorectal cancer and current treatments

Colorectal cancer (CRC) is among the most prevalent malignancies of the digestive tract, ranking third globally in incidence and second in cancer-related mortality [1]. It is a highly heterogeneous malignancy with distinct histopathological, molecular, and clinical characteristics. Histopathologically, early-stage CRC is confined to the mucosal or submucosal layers and presents in 2 primary morphological forms: polypoid and flat-elevated types. As CRC progresses, it adopts more aggressive invasion patterns, classified as exophytic (cauliflower-like), ulcerative (craterlike), and infiltrative types. At the molecular level, CRC pathogenesis follows 3 principal pathways: the adenoma–carcinoma sequence, the serrated pathway, and the inflammatory pathway, each contributing to tumor initiation and progression through distinct molecular mechanisms [2]. These pathways drive key genetic and epigenetic alterations that define CRC’s molecular subtypes, including microsatellite instability, chromosomal instability with adenomatous polyposis coli mutations, and oncogenic drivers such as Kirsten rat sarcoma virus, v-RAF murine sarcoma viral oncogene homolog B, and caudal type homeobox 2 alterations [3]. Given this molecular heterogeneity, the therapeutic strategy for CRC is highly personalized; a multidisciplinary approach is frequently adopted, incorporating endoscopic interventions, surgical resection, radiotherapy, chemotherapy, targeted therapy, and immunotherapy in a tailored manner [4].

### Oral–gut migration of *Fusobacterium nucleatum*

**Fig. 1.**
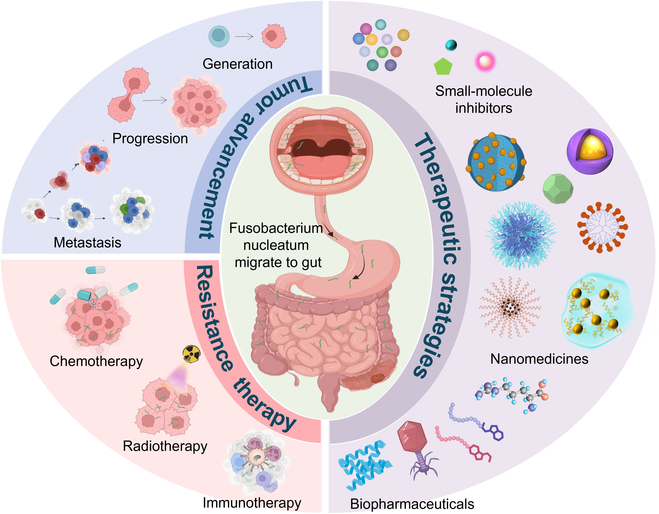
*Fusobacterium nucleatum* (*Fn*), which resides in the human oral cavity, exhibits a remarkable migratory capacity to the gut. When mislocalized in the gut, *Fn* profoundly contributes to the generation, progression, and metastasis of colorectal cancer (CRC), as well as induces resistance to chemotherapy, radiotherapy, and immunotherapy. Several therapeutic strategies have been proposed to target *Fn*, including small-molecule inhibitors, nanomedicines, and biopharmaceuticals.

*Fusobacterium nucleatum* (*Fn*) is a Gram-negative anaerobic bacterium commonly residing in the human oral cavity. It produces virulence factors that enhance its pathogenic potential, contributing to the development and progression of oral diseases such as odontogenic abscesses and periodontitis [5]. *Fn* exhibits a strong migratory capacity, enabling it to translocate from the oral cavity to the gut (Fig. 1), and has garnered considerable attention for its pivotal role in CRC pathogenesis. Studies have demonstrated that the oral and gut microbiota of CRC patients share comparable co-abundance networks, suggesting a potential ecological and evolutionary connection between these 2 microbial habitats [6]. Importantly, identical *Fn* strains have been isolated from both the colorectal tumor tissues and saliva samples of the same CRC patients, indicating that the *Fn* in colorectal tissues originates from the oral cavity [7]. Such evidence underscores the migratory behavior of *Fn* and its ability to maintain viability and pathogenicity across distinct niches.

### The epidemiology of *Fn*-related CRC

Clinical studies have demonstrated that intratumoral levels of *Fn* are substantially elevated in CRC patients compared to those in normal tissues [8]. A high abundance of *Fn* is strongly associated with poorer patient prognosis and increased cancer recurrence [9,10]. Quantitative polymerase-chain-reaction-based analysis has revealed an inverse correlation between intratumoral *Fn* levels and CRC survival. Compared to *Fn*-negative cases, the hazard ratio for CRC-specific mortality was 1.25 in *Fn*-low cases and 1.58 in *Fn*-high cases [11]. These findings suggest that modulating *Fn* abundance could be a potential strategy for CRC treatment, offering new therapeutic avenues to improve patient outcomes.

### *Fn* promotes the generation, progression, and metastasis of CRC

**Fig. 2.**
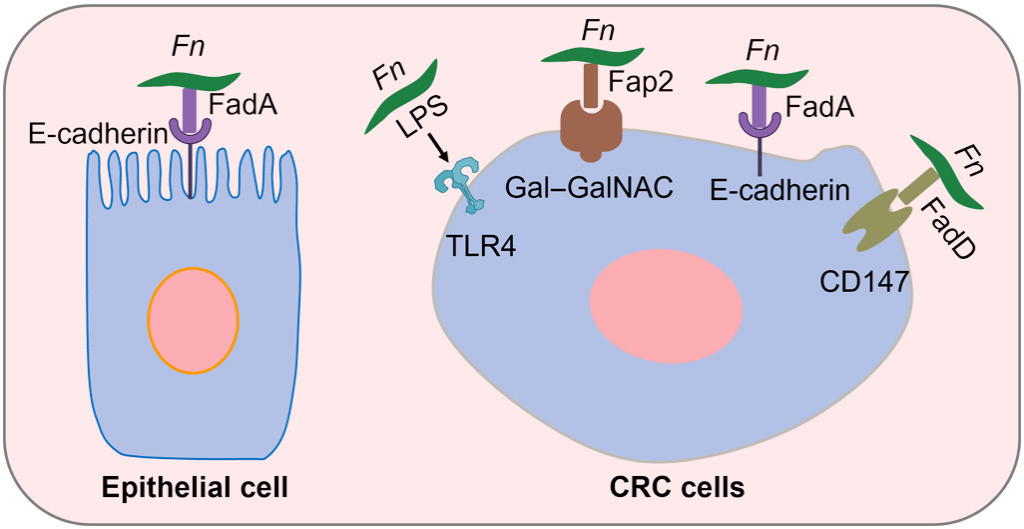
The major potential molecular mechanisms of *Fn* in CRC carcinogenesis. Gal, galactose; GalNAc, *N*-acetylgalactosamine; TLR4, Toll-like receptor 4.

*Fn* employs a repertoire of virulence factors, including FadA, Fap2, and RadD, as well as toxins such as lipopolysaccharides, which contribute to its pathogenicity and role in tumor biology [12]. Here, we first discuss the carcinogenic factors and the potential molecular mechanisms of *Fn* in CRC (Fig. 2). The primary pathogenic mechanism involves FadA-mediated cellular interactions, where this unique fusobacterial adhesin facilitates both adherence to and invasion of host epithelial and endothelial cells. This process disrupts endothelial integrity by increasing vascular permeability through junctional complex destabilization, enabling bacterial translocation across the endothelial barrier [13]. Afterward, FadA activates the E-cadherin/β-catenin signaling pathway, thereby stimulating the growth of CRC cells [14]. Under pathological conditions, the amyloid form of FadA enhances acid tolerance, further supporting *Fn*’s gastrointestinal migration and colonization [15]. As CRC progresses, Fap2, a galactose (Gal)-specific lectin, mediates selective tumor colonization through specific interactions with Gal–*N*-acetylgalactosamine (GalNAc) moieties that are abundantly expressed on malignant cells, thereby amplifying tumor progression [16]. Complementing these mechanisms, RadD facilitates *Fn* tumor colonization by interacting with the highly expressed receptor protein CD147 on CRC cells, contributing to *Fn*’s pathogenic potential by orchestrating tumor microenvironment modifications that favor bacterial persistence and tumor growth [17]. Furthermore, *Fn*-derived adhesins demonstrate specific binding affinity for RNA helicase family proteins expressed on CRC cells, establishing additional molecular bridges between bacterial colonization and tumorigenesis [18]. In addition to adhesins, the lipopolysaccharides of *Fn* could activate the β-catenin signaling pathway, subsequently engaging the Toll-like receptor 4/p21-activated kinase 1 cascade in CRC cells, thereby accelerating tumor progression [19]. Collectively, these processes of bacterial adherence, invasion, and colonization enable tumorigenesis and progression.

In advanced stages, *Fn* is not only highly enriched in primary CRC sites but also capable of migrating to distant metastatic locations [20]. *Fn* directly enhances the metastatic potential of CRC cells through multiple molecular pathways. *Fn* enhances CRC metastasis by promoting tumor–endothelial adhesion through activation of the alpha kinase 1/nuclear factor kappa-light-chain-enhancer of activated B cells/intercellular adhesion molecule 1 axis, thereby facilitating extravasation and metastatic dissemination [21]. Moreover, *Fn* inhibits methyltransferase-like 3-mediated N6-methyladenosine RNA modification, a critical regulatory mechanism in tumor progression, further contributing to metastasis [22]. Beyond its direct effects on tumor cells, *Fn* orchestrates tumor microenvironment remodeling by recruiting myeloid-derived immune cells to infection sites and promotes pro-invasive transcriptional reprogramming in CRC epithelial cells, thereby promoting metastatic progression [23]. These findings highlight the multifaceted role of *Fn* in CRC pathogenesis and its potential as a promising therapeutic target for mitigating disease progression and metastasis.

### *Fn* is associated with resistance to chemotherapy, radiotherapy, and immunotherapy in CRC

Clinical evidence has established a strong correlation between elevated *Fn* abundance and adverse clinical outcomes in CRC patients, including reduced overall survival and increased recurrence rates [11]. During CRC treatment, *Fn* is associated with resistance to chemotherapy, radiotherapy, and immunotherapy, considerably complicating disease management. One mechanism by which *Fn* drives chemotherapy resistance is through the induction of autophagy in CRC cells. Specifically, *Fn* promotes CRC cell survival by activating autophagy-related signaling pathways, thereby reducing the effectiveness of chemotherapeutic agents like 5-fluorouracil and oxaliplatin [24]. Wang et al. [25] demonstrated that *Fn*-induced chemoresistance in CRC is mediated, at least in part, through suppression of the Hippo signaling pathway, which protects tumor cells from chemotherapy-induced pyroptosis. Beyond chemotherapy, *Fn* also impaired the tumoricidal effects of radiation therapy and elicited CRC radioresistance [26]. The influence of *Fn* extends to immunotherapy resistance, actively shaping the development of an immunosuppressive tumor microenvironment. A key mechanism involves *Fn*-mediated recruitment of tumor-associated macrophages that exhibit elevated programmed death-ligand 1 expression, effectively suppressing T-cell-mediated antitumor immunity through immune checkpoint activation [27]*.* Furthermore, *Fn*-derived metabolic byproducts, particularly succinate and formate, play crucial roles in modulating local immune responses. These metabolites have been shown to alter immune cell function and polarization, ultimately contributing to diminished responses to immune checkpoint inhibitors and other immunotherapeutic approaches [28,29]. Collectively, these findings highlight the multifaceted role of *Fn* in conferring therapeutic resistance across multiple treatment modalities, underscoring the need for comprehensive strategies to overcome *Fn*-mediated treatment barriers in CRC management.

## Various Therapeutic Strategies Targeting *Fn* for CRC

Given that *Fn* has a deep impact on the pathogenesis of CRC, targeting and eliminating *Fn* to disrupt its carcinogenic activity holds considerable potential for reducing its contribution to tumor progression and enhancing the efficacy of existing cancer treatment regimens. A variety of strategies have been proposed, spanning modalities such as small-molecule inhibitors, nanomedicines, and biopharmaceuticals. The following sections delve into these approaches.

### Small-molecule inhibitors and delivery systems

Antibiotics are widely used antimicrobial agents, and most clinical isolates of *Fn* are sensitive to antibiotics such as metronidazole, clindamycin, and various β-lactam antibiotics. For example, metronidazole has been shown to reduce *Fn* burden in mice bearing patient-derived CRC xenografts, thereby inhibiting tumor growth [20]. Interestingly, beyond antibiotics, aspirin (a nonsteroidal anti-inflammatory drug) [30] and lauric acid (a fatty acid) [31] can also reduce *Fn* abundance in CRC tissues. While small-molecule inhibitors offer a straightforward approach to eliminating *Fn*, their use is often associated with low bioavailability and disruption of beneficial gut microbiota. To address these limitations, advanced delivery systems have been developed. For instance, Wang et al. [32] developed liposome-encapsulated antibiotics, which specifically target *Fn* and preserve the broader microbiome. Liu et al. [33] engineered an *Fn*-derived outer-membrane-vesicle-coated nanoplatform delivering metronidazole, which precisely targets tumor tissues. Additionally, a tunable nanogel was developed to enable the cascade release of metronidazole and chemotherapeutic agents [34], and a targeted polymer was also designed to efficiently deliver lauric acid [35], with both delivery systems enhancing therapeutic outcomes against *Fn*-associated CRC. However, the use of small-molecule inhibitors inevitably disrupts the gut microbiota beyond *Fn*, potentially eliminating commensal bacteria with tumor-suppressive properties, which constitutes a substantial therapeutic limitation.

### Nanomedicines

Nanomedicine-based antimicrobial approaches have shown strong capabilities in targeting and combating *Fn*. Au@BSA-CuPpIX nanoparticles effectively target *Fn* within tumors, generating reactive oxygen species under ultrasound, reducing anti-apoptotic protein levels, and promoting apoptosis [36]. Similarly, BSA-Cu single-atom nanozyme catalyzes reactive oxygen species production to eliminate *Fn*, suppress autophagy, and induce cancer cell apoptosis, disrupting the pathogen–tumor symbiosis [37]. Yan et al. [38] designed a positively charged polymer, polyamidoamine–lauric acid–cyclodextrin, with *Fn* inhibition capacity, specifically for the treatment of drug-resistant CRC. Additionally, a 2-dimensional metal–organic framework-based nanocomposite exhibits efficient antimicrobial activity against *Fn* [39]. Furthermore, Chen et al. [40] designed a nitroreductase-triggered supramolecular assembly technique, which selectively inhibits *Fn* and other cancer-promoting bacteria, thereby improving CRC treatment outcomes. Another novel strategy involves a GalNAc-derived nanomaterial that functions as a bacterial adhesion antagonist. This nanomaterial leverages GalNAc exposure to effectively inhibit *Fn* attachment to CRC cells through a Fap2-dependent competitive mechanism [41]. These studies underscore the versatility of nanomaterials in specifically targeting or combating *Fn*, offering innovative strategies to control its pathogenic effects and enhance CRC therapies.

### Biopharmaceuticals

Antimicrobial peptides (AMPs) show promise in CRC treatment due to their ability to kill *Fn* by disrupting bacterial membranes. However, their short half-life has hindered clinical applications. To overcome this limitation, modified AMP derivatives have been developed; the derivatives specifically target the membrane-associated protein FadA to disrupt the *Fn* membrane, thereby suppressing *Fn* activity and inhibiting *Fn*-induced CRC growth [42]. Although AMPs exhibit certain species selectivity, they lack the precision required for targeted bacterial elimination. In contrast, phage therapy, with its strain-specific targeting capability, represents a superior strategy for the selective eradication of *Fn*. Zheng et al. [43] isolated a phage from human saliva capable of lysing *Fn* and developed a nanocarrier system combining irinotecan with phages to target *Fn*-enriched CRC tumors. Additionally, M13 phages conjugated with silver nanoparticles (M13@Ag) effectively eliminate *Fn*, and this strategy substantially improved survival outcomes in CRC mouse models [44].

## Future Therapeutic Strategies Specifically Targeting *Fn* in CRC

Despite these therapies demonstrating potential for targeted *Fn* clearance in CRC treatment, most of these therapeutic strategies remain in the early stages of development and have inherent limitations. As the concept of targeting tumor-associated *Fn* in cancer therapy is still evolving, the development of new strategies, pathways, and targets to overcome these limitations will be essential for enhancing the applicability of treatments.

### Accurately targeted *Fn* subspecies

*Fn* subspecies adapt to the tumor niche through distinct genetic traits, and their dominance in promoting cancer progression makes specific evolutionary branches of *Fn* promising therapeutic targets. Studies show that *Fn* subspecies have historically been considered largely interchangeable in laboratory and clinical research. This misconception arises partly from the limitations of conventional 16S sequencing methods, as the 16S ribosomal RNA genes of *Fn* subspecies exhibit extensive sequence similarity [45]. Among these subspecies, pangenome analyses indicate active expression of *Fusobacterium animalis* (*Fna*) genes in CRC tumors, particularly within the mesenchymal subtype [46]. Recent genomic analyses challenge the notion of *Fna* as a single homogenous subspecies, revealing that *Fna* comprises 2 distinct clades: *Fna C1* and *Fna C2*. Excitingly, *Fna C2* is the dominant clade in the CRC tumor niche [47]. At present, eliminating *Fn* subspecies is an underexplored area in further research. This deeper understanding of *Fn* subspecies stratification, particularly the role of *Fna* and its clades, highlights the potential for developing tailored therapeutic strategies targeting specific *Fn* subspecies to more effectively mitigate their impact on cancer progression.

### Accurately targeted intracellular *Fn*

The intratumoral bacteria are predominantly intracellular, localized within both cancer cells and immune cells [48]. While vaccination can induce specific immune responses, some *Fn* evade immune clearance by invading tumor cells. A promising strategy involves developing novel approaches to target intratumoral *Fn*, disrupting their ability to hide and survive within host cells. At present, Meng et al. [49] described the use of cationic polymers to selectively cap phage heads, which remain intact and inhibit intracellular pathogenic bacteria. Similarly, Bai et al. [50] reported an oligoguanidine-based peptidomimetic that effectively targets and eradicates intracellular *Staphylococcus aureus* persisters within the phagolysosome lumen. Since these methods have been applied to only a limited number of intracellular bacterial pathogens, further studies are needed to explore their potential as a preferred strategy for targeting *Fn*.

### Specific vaccines

Vaccine-based therapies represent a promising strategy for CRC treatment, enabling both specific *Fn* clearance through immune recognition of bacterial epitopes and effective elimination of intracellular bacterial reservoirs via induced cellular immunity. Live attenuated vaccines have been in use since the last century and remain a valuable approach in combating bacterial infections. In one study, mice immunized with ultraviolet-inactivated *Fn* showed a marked reduction in abscess progression, demonstrated by markedly decreased swelling of gingival pocket tissues. Notably, the immunized mice also produced neutralizing antibodies that effectively inhibited *Fn*’s ability to generate volatile sulfur compounds, further highlighting the potential of this vaccination strategy [51]. Additionally, an antimicrobial vaccine mimicking the characteristics of *Fn* membranes has been developed, effectively eliminating *Fn* within tumors while preserving the gut- and tumor-associated microbiota [52].

Targeting *Fn*-specific virulence factors or host cell interaction receptors presents both opportunities and challenges for developing precise and effective immunotherapies against *Fn*-associated CRC. Liu et al. investigated vaccines targeting *Fn*’s outer membrane protein FomA, originally studied in the context of halitosis, which have shown potential in eliciting immune responses. However, evidence supporting their efficacy in reducing CRC incidence remains limited [53]. Proteins such as RadD, Fap2, and FadA, which contain predicted T-cell epitopes, are promising candidate antigens for *Fn* vaccines. These antigens can elicit a T-cell response and have the potential to selectively eliminate *Fn* within the tumor microenvironment. Nevertheless, further research is needed to refine vaccine designs and optimize their effectiveness in mitigating *Fn*’s role in CRC progression. For instance, incorporating immunological adjuvants into *Fn* vaccines may enhance their therapeutic efficacy by prolonging antigen persistence, amplifying costimulatory signaling, and promoting antigen-specific immune responses.
